# Retinal artery occlusion as a probable idiosyncratic reaction to topical minoxidil: a case report

**DOI:** 10.1186/s13256-021-03114-8

**Published:** 2021-10-08

**Authors:** Ramesh Venkatesh, Arpitha Pereira, Nikitha Gurram Reddy, Naresh Kumar Yadav

**Affiliations:** Dept. of Retina-Vitreous, Narayana Nethralaya, 121/C, Chord Road, 1st ‘R’ Block, Rajaji Nagar, Bangalore, 560010 India

**Keywords:** Minoxidil solution, Ocular, Retinal artery occlusion, Side effects

## Abstract

**Background:**

Minoxidil hair formulation is commonly used for the treatment of male or female androgenic alopecia. This over-the-counter product is considered safe, but should be used with caution. Ocular side effects following topical minoxidil use are rarely reported. In this paper, we report a rare case of inferior hemiretinal artery occlusion possibly caused by topical 5% minoxidil treatment.

**Case description:**

A 21-year-old Asian Indian male presented to the retina clinic with sudden onset blurring of vision and superior visual field loss in the right eye since morning. He was diagnosed with androgenic alopecia and was on treatment with topical 5% minoxidil spray twice a day for the last 3 years. On examination, his corrected distance visual acuity was 6/6, N6 in both eyes. Anterior segment examination and intraocular pressure in both eyes and left eye fundus were within normal limits. Right eye fundus examination showed features suggestive of inferior hemiretinal artery occlusion, which were confirmed on fluorescein angiography and optical coherence tomography. A detailed systemic evaluation and investigations (blood pressure, random blood sugar, hematological and coagulation profile, serum homocysteine level, Mantoux test, chest x-ray, cardiac two-dimensional echography, thyroid function test, and immunological profile) did not detect any abnormalities. The ocular condition and its visual prognosis were explained to the patient, and he was asked to review after 4 weeks.

**Conclusion:**

Though there is no definite cause–outcome relationship between topical minoxidil use and retinal artery occlusion development, this possibility should be kept in mind when observing retinal vascular occlusion cases with concurrent use of topical minoxidil.

## Background

Minoxidil hair formulation is commonly used in the treatment of male or female androgenic alopecia. This drug is available in the market as a 2% and 5% topical solution [1]. Minoxidil is believed to stimulate hair growth by increasing the anagen phase of the hair cycle, but the exact mechanisms remain elusive [2]. This over-the-counter product is considered usually safe, but should be used with caution.

Furthermore, minoxidil is an orally active vasodilator for treatment of severe hypertension. Typical systemic side effects of minoxidil are increased heart rate, augmented heart function and stroke volume, sodium and water retention, and abnormal hair growth [3]. The most common adverse reactions of topical formulations are limited to irritant and allergic contact dermatitis on the scalp [4]. Ocular side effects following topical minoxidil usage have rarely been reported [5–7]. The side effects following minoxidil therapy are dependent on the contact time of applied dose, concentration, and percutaneous absorption of the topical solution [8]. Herein, we report a case of inferior hemicentral retinal artery occlusion most likely caused by topical 5% minoxidil treatment.

## Case presentation

**Fig. 1:**
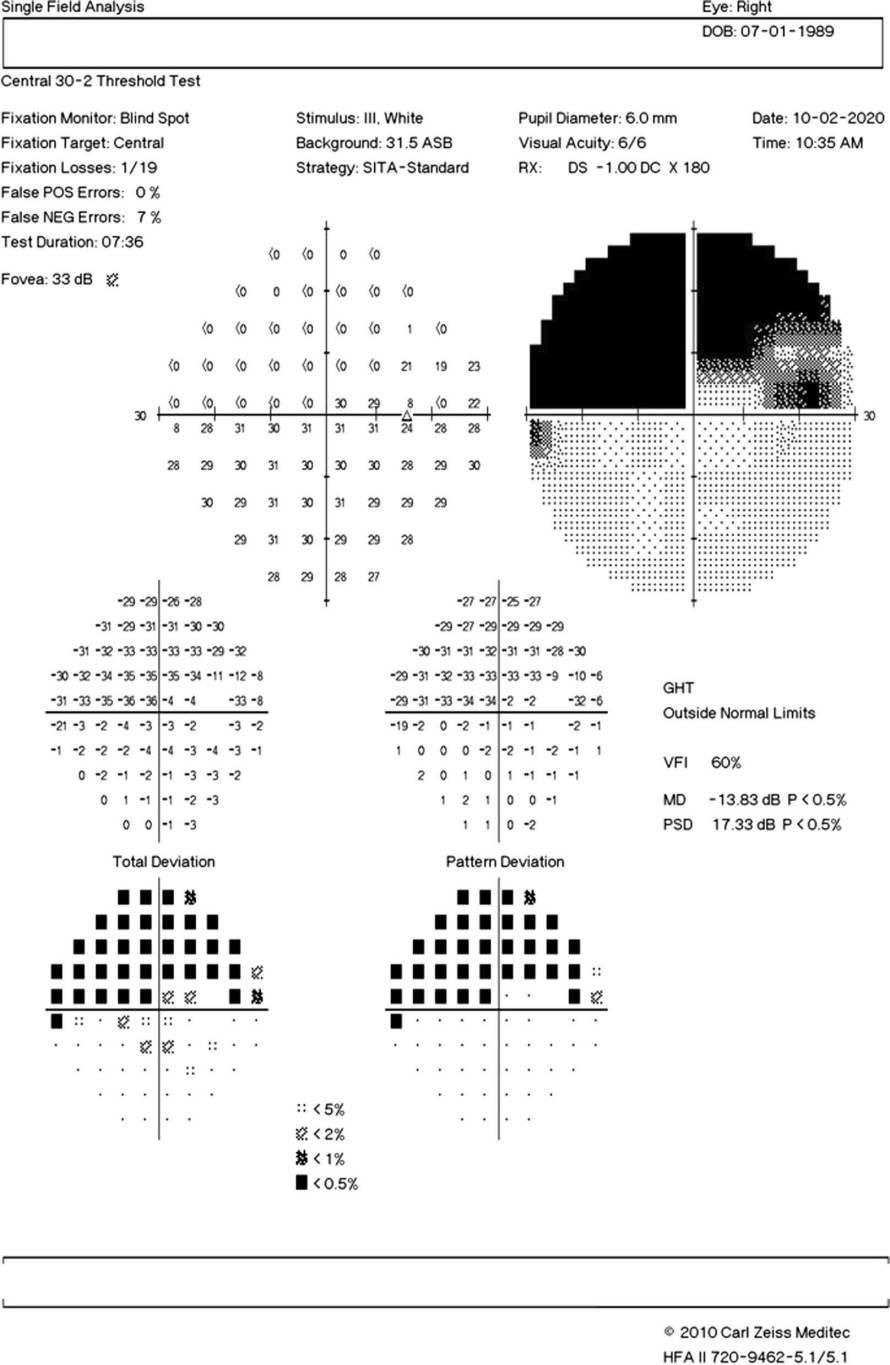
Humphrey visual field analysis of the right eye using the 30-2 protocol showing a superior visual field defect

**Fig. 2:**
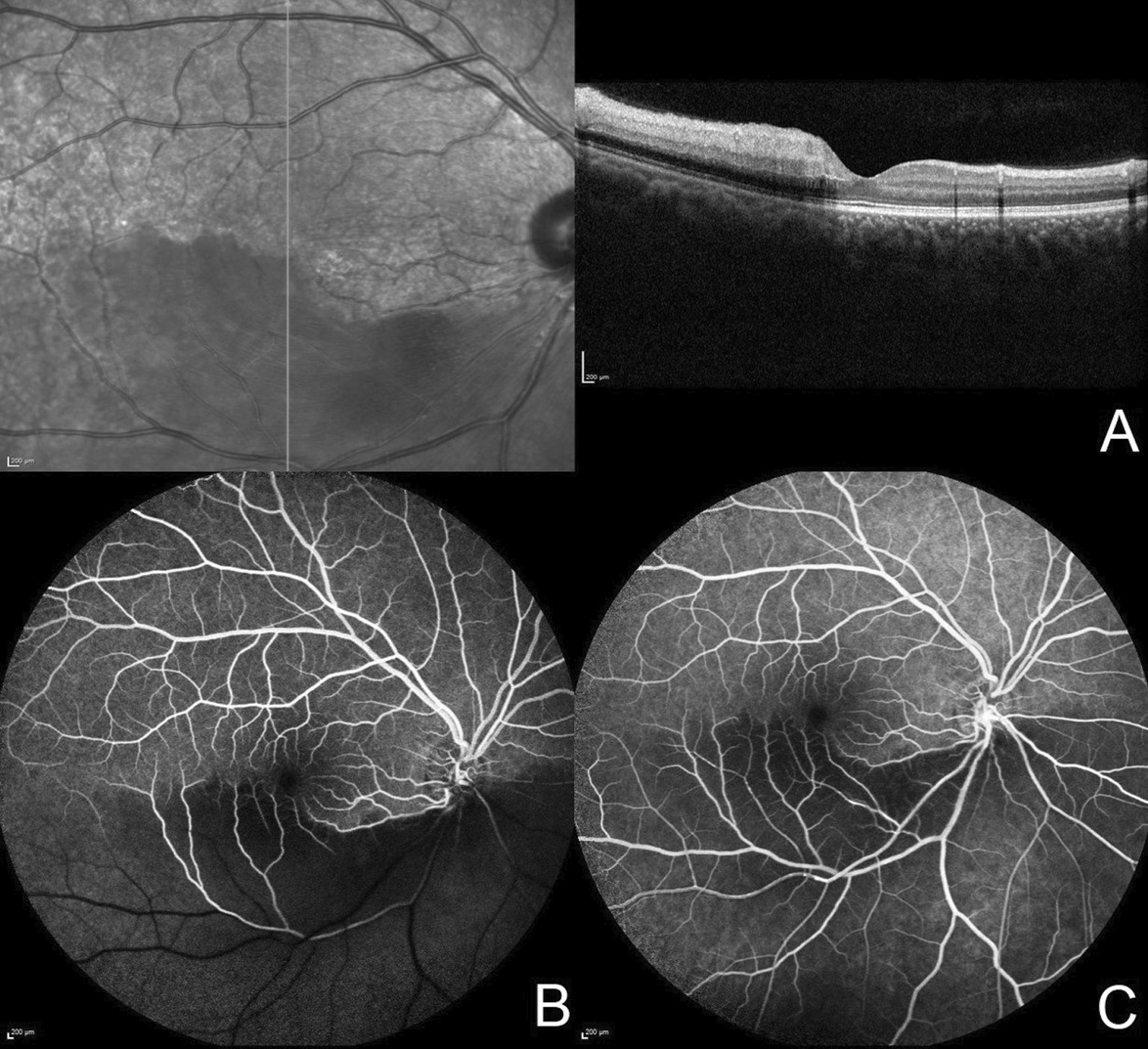
Fluorescein angiography and optical coherence tomography images in a case of inferior hemiretinal artery occlusion. **A** Spectral domain optical coherence tomography showing thickening and increased hyperreflectivity of inner retinal layers with hyporeflective outer retinal layers at the inferior macula. **B**, **C** Fundus fluorescein angiography of the right eye showing delayed filling of the inferior branch of the central retinal artery with corresponding blocked choroidal fluorescence due to retinal opacification

**Fig. 3:**
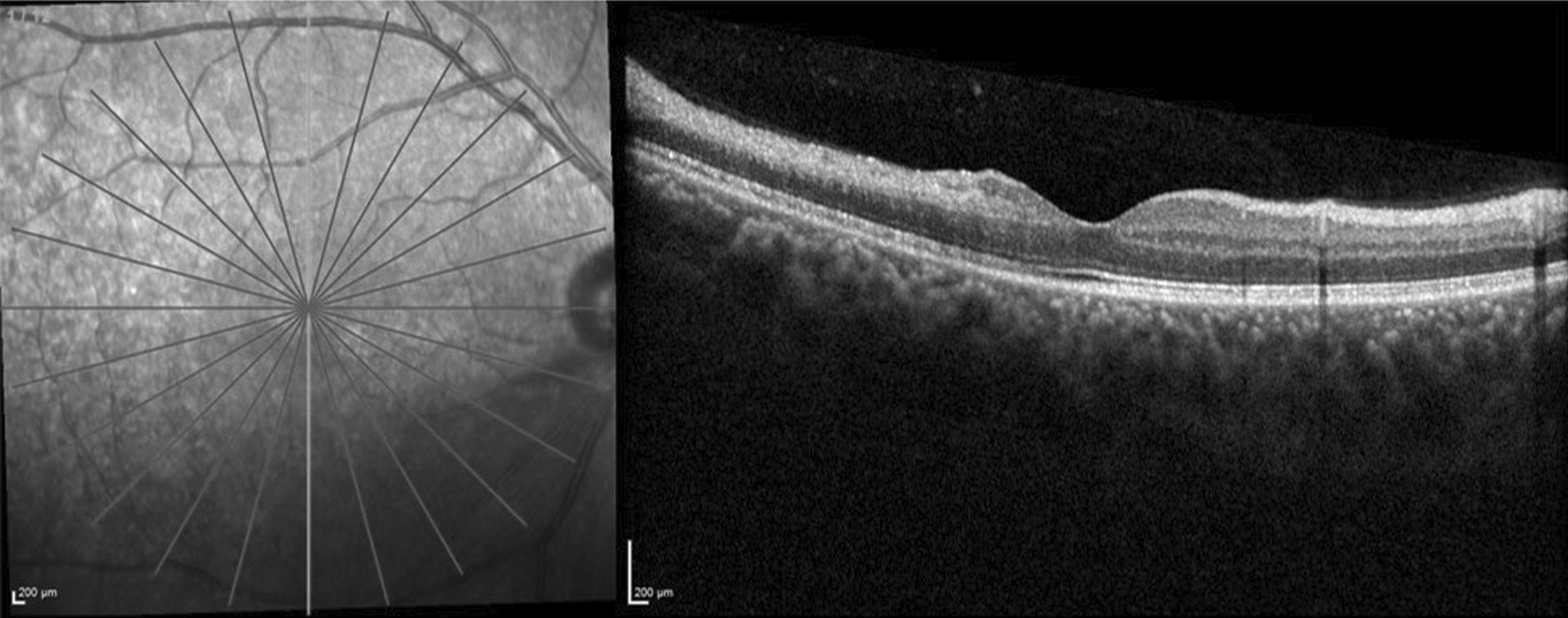
Optical coherence tomography (OCT) image in a case of inferior hemiretinal artery occlusion at 4 weeks post-presentation. Vertical line scan OCT image of the right eye at 4 weeks post-presentation in a case of inferior hemiretinal artery occlusion shows thinning and atrophy of the inner retinal layers inferior to the macula. The inner retinal layers superior to the macula show normal inner retinal thickness and retinal layer stratification

A 21-year-old Asian Indian male, non-smoker, presented to the retina clinic with sudden onset blurring of vision and superior visual field loss in the right eye since waking up that morning. The patient did not give history of similar episodes in the past. He had no other significant medical history other than alopecia. Family history was not significant for similar ocular or hair problems. He was treated for androgenic alopecia with topical 5% minoxidil spray (*Am-Exidil 5 topical solution*) twice a day for the last 3 years. Every application involved two puffs of spray directly applied to 40–45% of the scalp surface area. Examination of the scalp showed no signs of irritation, inflammation, or dermatitis at the time of initial presentation. On examination, his corrected distance visual acuity was 6/6, N6 in both eyes. Anterior segment examination and intraocular pressure in both eyes were within normal limits. The right eye fundus showed retinal opacification originating from the disc and spreading along the inferior arcade and involving the inferior macula with a normal foveal reflex. The left eye fundus was normal. A clinical diagnosis of inferior hemicentral retinal artery occlusion was made in the right eye. Humphrey 30-2 visual field testing showed a superior field defect (Fig. 1). Optical coherence tomography (SD-OCT, Spectralis HRA, Heidelberg Engineering, Heidelberg, Germany) of the right eye showed thickening and increased hyperreflectivity of inner retinal layers with hyporeflective outer retinal layers due to shadowing at the inferior macula (Fig. 2A). Fundus fluorescein angiography of the right eye (Spectralis HRA, Heidelberg Engineering, Heidelberg, Germany) revealed delayed filling of the inferior branch of the central retinal artery with corresponding blocked choroidal fluorescence due to retinal opacification (Fig. 2B, C). A detailed systemic evaluation and investigations (blood pressure, random blood sugar, hematological and coagulation profile, serum homocysteine level, Mantoux test, chest x-ray, thyroid function test, and immunological profile) did not detect any abnormalities. The values of the hematological and coagulation profile were as follows: red blood cell count 5.73 million/mm^3^, packed cell volume level 46.2%, mean corpuscular volume 80.6 fl, mean corpuscular hemoglobin 28.4 pg, mean corpuscular hemoglobin concentration 35.3 g/dL, red blood cell distribution width 12%, platelet count 2.00 lakhs/mm^3^, bleeding time 2 minutes 30 seconds, clotting time 5 minutes 30 seconds, and serum homocysteine level 8.65 µmol/L. Cardiac evaluation with two-dimensional (2D) echography was also found to be normal. No treatment was provided to the patient. The condition of the eye and its visual prognosis was explained to the patient, and he was asked to review after 1 week. After 1 week, his visual acuity was 6/6, N6 in both eyes. There was reduction in the retinal opacification. At 4 weeks, there was complete resolution of the inferior retinal opacification and thinning and atrophy of the inner retinal layers while the complaint of superior field defect still remained (Fig. 3). Written informed consent was obtained from the patient for including his clinical data and images in this report.

## Discussion and conclusion

A retinal artery occlusion occurs when the central retinal artery or one of its branches gets occluded leading to retinal opacification in the affected segment and visual field loss. Minoxidil sulfate is an active sulfated metabolite of minoxidil that causes opening of the K-ATP channels and vascular smooth muscle relaxation. In addition, minoxidil promotes the production of growth factors such as vascular endothelial growth factor (VEGF), and these might promote hair growth [2, 9–11]. Involvement of minoxidil in angiogenesis causes retinal capillary rearrangement and increased chances of vessel thrombosis and occlusion, which could have been the likely pathogenetic mechanism in this case [5]. Aktas *et al*. published a report of non-arteritic anterior ischemic optic neuropathy following short posterior ciliary artery occlusion due to high-dose and prolonged topical minoxidil usage in a young, otherwise healthy man [6]. Another non-peer-reviewed report showed retinal artery occlusion following minoxidil usage documented by a French physician in a 46-year-old lady being treated for alopecia [12]. Although retinal artery occlusion is thought to be associated with several other etiological factors such as diabetes, hypertension, coagulopathies, hyperlipidemia, and atherosclerosis, there was no known predisposing factor that could have caused a retinal artery occlusion in our patient, except for the high amount and long duration of use of topical minoxidil. The occurrence of retinal artery occlusion in an otherwise young healthy male suggests minoxidil could be the causative factor. On the basis of clinical history and ocular findings, Naranjo adverse drug reaction probability score was 5 [13], thus suggesting a probable idiosyncratic drug reaction to topical minoxidil usage. It is not possible to infer a direct relationship between topical minoxidil use and retinal artery occlusion development without prospective or retrospective studies. Thus, despite the use of minoxidil by the patient and the other online case report, the relationship between minoxidil and retinal artery occlusion still remains unproven. It is possible that this is only by chance.

To conclude, even though there is no definite cause–outcome relationship between topical minoxidil use and retinal artery occlusion development, this potential possibility should be kept in mind when observing retinal vascular occlusion cases with concurrent use of topical minoxidil. A larger collection of cases would be required to establish a cause–outcome relationship.

## Data Availability

The datasets used and/or analyzed during the current study are available from the corresponding author on reasonable request.

## Abbreviations

OCT: Optical coherence tomography
VEGF: Vascular endothelial growth factor
